# Oxidative Profile and δ-Aminolevulinate Dehydratase Activity in Healthy Pregnant Women with Iron Supplementation

**DOI:** 10.3390/ijerph13050463

**Published:** 2016-05-03

**Authors:** Leidiane De Lucca, Fabiane Rodrigues, Letícia B. Jantsch, Walter S. Neme, Francisco M. P. Gallarreta, Thissiane L. Gonçalves

**Affiliations:** 1 Postgraduate Program in Pharmaceutical Sciences, Department of Clinical and Toxicology Analysis, Center of Healthy Sciences, Federal University of Santa Maria (UFSM), Santa Maria 97105-900, Brazil; leidi_lucca@hotmail.com (L.D.L.); fabianeufsm@gmail.com (F.R.); letii_jantsch@hotmail.com (L.B.J.); 2 Departamet of Obstetrics and Gynecology, Federal University of Santa Maria (UFSM), Santa Maria 97105-900, Brazil; wsneme@yahoo.com.br (W.S.N.); fmgallarreta@msn.com (F.M.P.G.)

**Keywords:** iron, oxidative stress, antioxidant, δ-aminolevulinate dehydratase, pregnant women

## Abstract

An oxidative burst occurs during pregnancy due to the large consumption of oxygen in the tissues and an increase in metabolic demands in response to maternal physiological changes and fetal growth. This study aimed to determine the oxidative profile and activity of δ-aminolevulinate dehydratase (δ-ALA-D) in pregnant women who received iron supplementation. Oxidative stress parameters were evaluated in 25 pregnant women with iron supplementation, 25 pregnant women without supplementation and 25 non-pregnant women. The following oxidative stress parameters were evaluated: thiobarbituric acid reactive substances (TBARS), protein thiol groups (P-SH), non-protein thiol levels (NP-SH), vitamin C levels, catalase and δ-ALA-D activity. Markers of oxidative stress and cell damage, such as TBARS in plasma were significantly higher in pregnant women without supplementation. Levels of P-SH, NP-SH and δ-ALA-D activity were significantly lower in pregnant women without supplementation compared to non-pregnant and pregnant women with supplementation, while vitamin C levels were significantly lower in pregnant women without supplementation when compared to non-pregnant women. The increase in the generation of oxidative species and decrease of antioxidants suggest the loss of physiological oxidative balance during normal pregnancy, which was not observed in pregnant women with iron supplementation, suggesting a protective effect of iron against oxidative damage.

## 1. Introduction

Pregnancy is a physiological challenge to be met by the mother and fetus, because of the high metabolic demand and increased amount of oxygen in tissues, such as the placenta [1]. This increase in the oxygen demands, even in a normal pregnancy, is associated with an increase in the generation of reactive oxygen species (ROS) and, consequently, the presence of oxidative stress [2], defined as loss of balance between pro-oxidants and antioxidants [3]. This imbalance can occur due to a number of factors, such as excessive production of free radicals, overloading the amount of available antioxidants, leading to lowered defense against the harmful effects of free radicals [4,5].

The maintenance of homeostasis to the physiological functions depends on the balance between the generation of free radicals and antioxidant status of the organism. The antioxidants react rapidly with free radicals to prevent the progression of auto-oxidation, detoxify the excess ROS and thereby maintaining the balance between oxidants and antioxidants [6]. These antioxidant mechanisms include the enzymes catalase, glutathione peroxidase and superoxide dismutase [7] and exogenous antioxidants as vitamin C and vitamin E [4]. The sulfhydryl delta-aminolevulinate dehydratase enzyme (δ-ALA-D) is responsible for catalyzing the synthesis of tetrapyrrolic compounds, such as heme [8], which sulfhydryl groups in pro-oxidant conditions become susceptible to oxidation, leading to inhibition of enzyme. Inhibition of δ-ALA-D mainly affects heme biosynthesis, resulting in the accumulation of the substrate, 5-aminolevulinic acid (ALA), which contributes directly to ROS production and oxidative stress overall charge [9].

Oxidative stress can influence the entire reproductive period of a woman’s life and can play a significant role during pregnancy, normal childbirth and premature delivery [10]. During placental development, oxidative stress can reach excessive levels and is associated with various diseases during pregnancy [6].

Because of the high prevalence of iron deficiency during pregnancy [11] iron supplementation, especially in developing countries, is a fairly routine practice during gestation [12]. Maternal iron deficiency during pregnancy can compromise the development of the newborn brain and it may cause damage to physical and mental development [13]. In addition, Drevim *et al.* showed a worsening in oxidative stress related to iron deficiency in pregnant women [14].

In view of evidence highlighting increased oxidative stress during healthy pregnancies, the objective of the study was to verify the oxidative profile and the δ-ALA-D enzyme activity in pregnant women taking iron supplementation compared with pregnant women who were not taking supplementation and non-pregnant women in order to establish an understanding of this adaptation of the woman’s body to pregnancy and better understand the redox state of the reproductive phase of women.

## 2. Materials and Methods

### 2.1. Study Population

The study population consisted of healthy pregnant women receiving care at the basic health unit José Erasmo Crossetti in Santa Maria, Rio Grande do Sul, Brazil, and healthy non-pregnant women from the Federal University of Santa Maria, with 25 healthy pregnant women who were on iron supplementation (PIS) at concentrations of 40–120 mg/day, according to medical guidance, 25 healthy pregnant women without supplementation (PWS), and 25 non-pregnant healthy women (NP).

Healthy pregnant women were in their third trimester of pregnancy were included in the experimental group, while in the non-pregnant group, healthy women in the same age group were included. Pregnant and non-pregnant women with chronic conditions such as smoking, drinking, asthma, hypertension, preeclampsia, diabetes mellitus, infectious diseases, thyroid disease, cancer or any other chronic condition or that used medication or supplementation, with the exception of iron, were excluded due to the possibility of bias in the results.

Blood collections from the pregnant women were conducted every Tuesday from July 2015 to January 2016 at the prenatal clinic of the health center. The samples of non-pregnant women were collected in the same period. Pregnant women taking iron supplementation were those who had altered hemoglobin levels below 11 g/dL in the early months of pregnancy.

All participants gave written informed consent to participate in this study. The study was approved by the Human Ethical Committee of the Federal University of Santa Maria, under protocol number 22771613.6.0000.5346 and was in accordance with the Declaration of Helsinki (2000) of the World Medical Association. 

### 2.2. Sample Collection

Blood samples were collected by venipuncture, following fasting for 8 hours and the tests were processed immediately after blood collection. 16 mL were collected in vacuum tubes; 1 containing EDTA (4 mL) was used for a complete blood count and another containing sodium fluoride (4 mL) was used for the determination of glucose levels. Two tubes containing heparin anticoagulant (4 mL) were used to obtain whole blood, plasma and erythrocytes. The plasma was separated by centrifugation at 3000 rpm for 10 min and then the erythrocytes were washed three times with 0.9% NaCl.

### 2.3. Hematological, Biochemical and Clinical Parameters

Hematological parameters were evaluated with a Sysmex XE 5000 automated cell counter. Fasting blood glucose was measured in plasma by a standard method with a commercial kit (Bioclin, Minas Gerais, Brasil). Clinical parameters were also evaluated, including blood pressure of the participants, which was measured using an aneroid sphygmomanometer. The body mass index (BMI) was calculated by dividing weight by squared height (kg/m^2^).

### 2.4. Thiobarbituric Acid Reactive Substances

Lipid peroxidation in plasma was evaluated by measuring thiobarbituric acid reactive substances (TBARS) according to the method of Lapenna *et al.* [15] using 1% phosphoric acid and 0.6% thiobarbituric acid. The reaction product was measured spectrophotometrically at 532 nm and the results were expressed in n mol TBARS/mL of plasma.

Lipid peroxidation in red cells was also assessed by measuring TBARS according to the method of Lapenna *et al.* [15] using 1.2% thiobarbituric acid. The reaction product was measured spectrophotometrically at 532 nm and the results were expressed in nmol TBARS/mL of erythrocytes.

### 2.5. Protein Thiol Groups

Protein thiols groups (P-SH) were quantified in plasma by the method of Boyne and Ellman [16] modified by Jacques Silva *et al.* [17], which consists of reducing 5,5′-dithiobis(2-nitrobenzoic acid) (DTNB) in 0.3 M phosphate buffer (pH 7.0) measured at 412 nm. The results were expressed as nmol of P-SH/mL plasma.

### 2.6. Non-Protein Thiol Groups 

Erythrocyte non-protein thiols groups (NP-SH) were determined as described by Boyne and Ellman [16] and modified by Jacques Silva *et al.* [17]. The erythrocytes obtained after centrifugation of whole blood were hemolyzed with a 10% solution of triton for 10 min. Then, the protein fraction was precipitated with 20% trichloroacetic acid, followed by centrifugation. The colorimetric assay was performed in 1 M phosphate buffer (pH 7.4). The NP-SH levels were measured at 412 nm and expressed as nmol of NP-SH/mL erythrocytes.

### 2.7. Vitamin C

Vitamin C in plasma (VIT C) was evaluated as described by Galley *et al.* [18] with some modifications by Jacques-Silva *et al.* [17]. The precipitate was isolated fresh plasma with trichloroacetic acid solution 5%, followed by centrifugation. An aliquot of the supernatant was homogenized with 2,4-dinitrophenylhydrazine (DNPH) and 13.3% trichloroacetic acid followed by incubation for 3 h at 37 °C. Then, 1 mL of sulfuric acid 65% was added to the medium and the orange-red compound was measured at 520 nm. The ascorbic acid content was calculated using a standard curve (1.5–4.5 mmol/L of ascorbic acid freshly prepared in sulfuric acid) and expressed as µg VIT C/mL plasma.

### 2.8. Activity of Catalase

Catalase activity was determined as described by Aebi *et al.* [19], based on H_2_O_2_ decomposition, determined spectrophotometrically at 240 nm using H_2_O_2_ as substrate, with results expressed in K/mg·Hb.

### 2.9. δ-ALA-D Activity and Reactivity Index

δ-ALA-D activity was determined in whole blood by the method of Berlin and Schaller [20], measuring the formation rate of porphobilinogen in 1 h at 37 °C. The enzyme reaction was started after 10 min pre-incubation of blood by the addition of δ-ALA to a final concentration of 4 mM in a phosphate buffered solution. The reaction product was determined using a modified Ehrlich’s reagent at 555 nm with a molar absorption coefficient of 6.1 × 10^4^ M^−1^ for Ehrlich-porphobilinogen salt. Results were expressed in U/L (PBG nmol/h/mg·Hb). In order to determine whether changes in δ-ALA-D activity are related to the oxidation of thiol groups, a set of tubes was assayed using a similar incubation medium except that 2 mM of the reducing agent dithiothreitol (DTT) was added for the reactivation index. The reactivation index was estimated using: A−B/A × 100, where A = absorbance assay with DTT and B = without DTT absorbance assay.

### 2.10. Statistical Analysis

Data analysis was performed using the software Graph Pad Prism v.5. (Harvey Motulsky, San Diego, CA, USA). Shapiro-Wilk test was used to test the distribution of samples. The One-Way analysis of variance (ANOVA) was used for comparison between groups with normally distributed variables and the data were expressed as mean ± standard deviation (SD). When there was a statistically significant difference, the analysis was complemented by the Tukey test. Non-parametric results were determined by Kruskal-Wallis test and represented as median (interquartile range). A *p* value < 0.05 was considered statistically significant for all analyses.

## 3. Results

The clinical and demographic characteristics of the 75 pregnant and non-pregnant volunteer women who participated in this study are shown in Table 1.

There were no significant differences between groups in terms of age, height, gestational age, systolic pressure and diastolic pressure. However, as expected, there was a significant difference in weight and BMI between the group of non-pregnant women and the group of pregnant women with and without iron supplementation.

Hematological and biochemical parameters were analyzed, though some were not statistically significant between the groups of pregnant and non-pregnant women. However, other parameters were statistically significant between the groups as shown in Table 2.

With regard to hematological parameters, there was a significant decrease in erythrocytes, hematocrit and hemoglobin in the two groups of pregnant women when compared to the group of non-pregnant women, as can be seen in Table 2. On the other hand, there was no significant difference between the groups for glucose values and platelets.

With respect to oxidative stress markers, shown in Table 3, the TBARS levels in plasma were significantly higher in pregnant women without supplementation, whereas P-SH and NP-SH were significantly lower in pregnant women without supplementation when compared to pregnant women with iron supplementation and non-pregnant women, while vitamin C levels were significantly lower in pregnant women without supplementation when compared to non-pregnant women. TBARS in erythrocytes and catalase were not significantly different between pregnant and non-pregnant women. 

δ-ALA-D activity in the blood was significantly lower in pregnant women without supplementation when compared to non-pregnant women and pregnant women with supplementation, and the same was observed in the presence of DTT (Table 4). In addition, the enzyme reactivation index was higher in pregnant women without supplementation when compared to non-pregnant and pregnant women with supplementation (Table 4).

## 4. Discussion

In normal pregnancy there are physiological and anatomical adjustments that cause changes in the mother’s body, such as changes in hormones and in humoral and figurative elements of blood [21]. Usually there is a physiological anemia with decreased hemoglobin, due to increased plasma volume in relation to erythrocyte volume, resulting in a physiological blood dilution. During pregnancy, hematocrit usually ranges from 32% to 34% compared to non-pregnant women, and the transition from iron to the fetus may further aggravate this anemia. Due to platelet aggregation, the amount of platelets may be lower during pregnancy, even while it may remain within the reference values [22]. Hematological data of the volunteers in this study corroborate those from the literature, as shown in Table 2.

Some studies suggest that contact of cell membranes with ROS can lead to lipid peroxidation, promoting cell damage due to changes in the physical properties and structural organization of membrane components, resulting in loss of ion exchange selectivity, release of organelle content, such as lysosomal hydrolytic enzymes, and production of cytotoxic products, including malondialdehyde [23,24,25]. During normal pregnancy, lipid peroxidation is higher when compared with non-pregnancy in healthy women [26], which was confirmed in our study by increased levels of TBARS in plasma (Table 3), suggesting an increase of lipid membrane damage in pregnant women without supplementation when compared to non-pregnant women.

The thiol functional group (-SH) present in biomolecules performs a significant role in various physiological functions and pathological conditions by reducing properties, chelation of protein and protecting against oxidative stress, being susceptible to oxidative damage [27,28]. This study showed a decrease of thiol groups in plasma and erythrocytes of pregnant women without supplementation (Table 3) which may indicate lowered defense against oxidative damage. 

Vitamin C was significantly lower in the pregnant women without supplementation when compared to non-pregnant women (Table 3). This result is consistent with data from other studies suggesting that in pregnant women, ascorbate may be used to compensate for the disturbance in cells mediated by free radicals to maintain normal homeostasis during pregnancy [29,30].

The δ-ALA-D enzyme is the second enzyme in the heme biosynthetic pathway used to maintain the hemoglobin content of erythrocytes [31]. Its inhibition leads to the accumulation of ALA in the blood, which is related to the overproduction of ROS [32]. ALA undergoes autoxidation at physiological pH resulting in the formation of O_2_^−^, H_2_O_2_ and ALA free radicals [33], causing oxidative DNA damage, lipid peroxidation and depletion of the cellular antioxidant defense system [34]. The reduction of δ-ALA-D activity observed in pregnant women without supplementation in the present study (Table 4) suggests that inhibition of this enzyme may contribute to the overall levels of ROS in these pregnant women which is in accordance with the study by Ademuyiwae *et al.* [35], who found similar results, which may also be related to lower hemoglobin and hematocrit values. Furthermore, studies have shown that this enzyme protein is a marker of oxidative stress situations [9,36].

The analysis of in vitro activity of δ-ALA-D was performed by incubation with dithiothreitol, which is a reducing agent used both to reverse and to prevent oxidation of thiol groups (-SH) of the enzyme [37]. An index of greater enzymatic reactivation was observed in pregnant women without supplementation when compared to non-pregnant women and pregnant women with supplementation (Table 4), showing a possible involvement of the groups (SH), through partial restoration of δ-ALA-D activity in the presence of DTT. The portion that was not restored may be related to the oxidation of other groupings or even due to a decrease in its biosynthesis.

Iron deficiency during pregnancy mainly occurs due to inadequate dietary intake in this period where there is a greater need for this nutrient [38]. Despite the wide use of iron supplementation during pregnancy due to the high incidence of iron deficiency anemia, few studies have evaluated the effect of supplementation to the mother and the neonate, however, its use has been shown to improve hematological indices as noted in the study of Lunardi-Maia *et al.* [11].

Iron, used in low doses, is an essential mineral for cellular homeostasis and a cofactor in numerous biological reactions due to its ability to donate and receive electrons. In addition, it participates in the formation of several proteins and is essential for the formation of heme. When in the form of hemeprotein, it is essential for transporting oxygen, detoxification and energy generation [39]. Mitochondria are critical for iron metabolism because they are where the biosynthesis of Fe-S clusters and heme biosynthesis occur. Iron intake in the mitochondria is not yet fully understood. Frataxin, a protein which is located in the matrix and in the inner mitochondrial membrane, regulates iron utilization after being transported through the mitochondrial membrane, allocating it to the genesis of Fe-S clusters or to the synthesis of heme. By forming a complex with iron, frataxin prevents the formation of free radicals in the mitochondrion [40].

Iron supplementation during pregnancy is a conflicting subject in the literature. Some studies have shown that iron, in excess, can lead to increased oxidative stress [41,42], harming the metabolism of pregnant women and leading to complications, such as preeclampsia [43] and Gestational Diabetes Mellitus [44]. In contrast, other studies have suggested that iron is one of the most important micronutrients able to trap free radicals [45]. Iron supplementation can improve the hematological system, helping to normalize hematocrit and hemoglobin and to reduce the state of oxidative stress in anemic pregnant women. Considering the decline of some oxidative stress parameters caused due to anemia [46,47], it is a mineral which may act indirectly as a non-enzymatic antioxidant and thus an important cofactor of antioxidant enzymes [48]. This was confirmed in this study, where there was an improvement in the state of oxidative stress in pregnant women receiving supplementation with iron as shown in Table 3, through the reduction in plasma TBARS levels, along with increased levels of antioxidants such as thiol groups both in plasma and in the erythrocytes and an increase in δ-ALA-D activity.

## 5. Conclusions

The loss of normal physiological balance between generation of oxidative species and antioxidant capacity in the maternal organism during pregnancy, observed by increased oxidative substances, by falling antioxidant levels and inhibition of δ-ALA-D enzyme activity, suggests an increased oxidative profile in pregnant women who do not use iron supplementation when compared with pregnant women who are using supplementation and non-pregnant women. Iron supplementation may promote a protective effect against oxidative damage produced during pregnancy. However, other studies involving a larger number of patients are needed to confirm these findings.

## Figures and Tables

**Table 1:** Clinical and demographic characteristics of pregnant and non-pregnant women.

Characteristics of Subjects	NP (*n* = 25)	PWS (*n* = 25)	PIS (*n* = 25)
Age (years)	26.50 (23.75–29.00)	26.00 (22.25–30.00)	23.00 (20.00–28.00)
Height (cm)	164.80 ± 6.08	161.90 ± 5.48	160.70 ± 6.50
Weight (kg)	60.00 (56.00–68.25)	78.00 (67.50–90.77) ^1^	73.20 (66.10–87.48) ^1^
BMI (kg/m²)	22.23 (21.17–24.86)	28.06 (26.87–34.55) ^1^	28.70 (26.60–32.59) ^1^
Gestational age (weeks)	-	32.94 ± 3.13	33.55 ± 2.85
Systolic pressure (mmHg)	110.00 (110.00–120.00)	110.00 (102.50–120.00)	110.00 (100.00–110.00)
Diastolic pressure (mmHg)	70.00 (60.00–80.00)	70.00 (70.00–77.50)	70.00 (60.00–70.00)

Parametric results were determined by ANOVA followed by Tukey test and represented as mean ± standard deviation and non-parametric results were determined by Kruskal-Wallis and represented as median (interquartile range). ^1^ *p* < 0.05 when compared with the group of non-pregnant women; NP: non-pregnant women; PWS: pregnant women without supplementation with iron; PIS: pregnant women with iron supplementation.

**Table 2:** Hematological and biochemical parameters of pregnant and non-pregnant women.

Parameter	NP (*n* = 25)	PWS (*n* = 25)	PIS (*n* = 25)
Erythrocytes (10^6^/mm³)	4.66 ± 0.31	3.91 ± 0.24 ^1^	4.13 ± 0.30 ^1^
Hematocrit (%)	40.85 (38.60–43.55)	35.00 (33.93–36.90) ^1^	36.40 (35.20–38.15) ^1^
Hemoglobin (g/dL)	13.25 (12.60–14.07)	11.60 (11.23–12.25) ^1^	11.90 (11.43–13.20) ^1^
Platelets (MIL/mm³)	248.50 (215.50–282.80)	239.00 (191.50–263.80)	218.00 (203.00–241.00)
Glucose (mg/dL)	77.88 ± 5.43	74.38 ± 6.60	75.90 ± 6.93

Parametric results were determined by ANOVA followed by Tukey test and represented as mean ± standard deviation and non-parametric results were determined by Kruskal-Wallis and represented as median (interquartile range).^1^ *p* < 0.05 when compared with the group of non-pregnant women; NP: non-pregnant women; PWS: pregnant women without supplementation with iron; PIS: pregnant women with iron supplementation

**Table 3:** Oxidative stress markers in pregnant and non-pregnant women.

Parameter	NP (*n* = 25)	PWS (*n* = 25)	PIS (*n* = 25)
TBARS plasma (nmol/mL)	3.45 ± 1.39	4.86 ± 1.47 ^1,2^	3.59 ± 1.37
TBARS erythrocytes (nmol/mL)	13.37 ± 4.86	14.13 ± 4.96	15.71 ± 4.96
P-SH (nmol P-SH/mL)	149.60 ± 14.40	128.90 ± 22.34 ^1,2^	150.10 ± 20.66
NP-SH (nmol NP-SH/mL)	927.90 ± 163.40	694.90 ± 150.40 ^1,2^	829.50 ± 155.90
VITAMIN C (μgvit C/mL)	18.94 ± 5.69	14.30 ± 5.87 ^1^	14.83 ± 6.18
CATALASE (K/mg·Hb)	49.32 ± 7.65	48.04 ± 8.04	53.85 ± 9.01

Data expressed as mean ± standard deviation. The statistically significant differences were determined by ANOVA followed by Tukey test. ^1^ *p* < 0.05 for comparisons with the group of non-pregnant women; ^2^ *p* < 0.05 when comparing with the group of pregnant women with iron supplementation; NP: non-pregnant women; PWS: pregnant women without supplementation with iron; PIS: pregnant women with iron supplementation. TBARS: thiobarbituric acid reactive substances, P-SH: thiol groups of proteins in the plasma, NP-SH: non-protein thiol groups in erythrocytes.

**Table 4:** Delta-aminolevulinate dehydratase activity and Reactivation Index in pregnant and non-pregnant women.

Parameter	NP (*n* = 25)	PWS (*n* = 25)	PIS (*n* = 25)
δ-ALA-D (U/L)	56.67 (46.66–68.73)	41.18 (26.36–45.73) ^1,2^	47.67 (65.67–39.21)
δ-ALA-D + DTT (U/L)	73.70 (63.91–86.27)	53.54 (41.05–61.70) ^1,2^	65.34 (59.98–84.04)
Reactivation Index (%)	16.10 (13.17–27.00)	31.84 (29.39–34.71) ^1,2^	24.30 (21.50–29.30)

Data expressed as median (interquartile range). Statistically significant differences were determined by Kruskal-Wallis. ^1^ *p* < 0.05 when compared with the group of non-pregnant women; ^2^ *p* < 0.05 when compared with the group of pregnant women with iron supplementation; NP: non-pregnant women; PWS: pregnant women without supplementation with iron; PIS: pregnant women with iron supplementation; δ-ALA-D: delta-aminolevulinate dehydratase; DTT: dithiothreitol.

## Abbreviations

The following abbreviations are used in this manuscript:

ALA	5-aminolevulinic acid
BMI	body mass index
DTT	dithiothreitol
NP	non-pregnant women
NP-SH	non-protein thiol groups
PIS	pregnant women who were on iron supplementation
P-SH	Protein thiol groups
PWS	pregnant women without supplementation
ROS	reactive oxygen species
SD	standard deviation
TBARS	thiobarbituric acid reactive substances
VIT C	vitamin C
δ-ALA-D	delta-aminolevulinate dehydratase

## References

[1] Aversa S., Pellegrino S., Barberi I., Reiter R.J., Gitto E. (2012). Potential utility of melatonin as an antioxidant during pregnancy and in the perinatal period. J. Matern. Fetal Neonatal Med..

[2] Gitto E., Pellegrino S., Gitto P., Barberi I., Reiter R.J. (2009). Oxidative stress of the newborn in the pre-and postnatal period and the clinical utility of melatonin. J. Pineal Res..

[3] Al-Gubory K.H., Fowler P.A., Garrel C. (2010). The roles of cellular reactive oxygen species, oxidative stress and antioxidants in pregnancy outcomes. Int. J. Biochem. Cell. Biol..

[4] Burton G.J., Jauniaux E. (2011). Oxidative stress. Best Pract. Res. Clin. Obstet. Gynaecol..

[5] Balsano C., Alisi A. (2009). Antioxidant effects of natural bioactive compounds. Curr. Pharm. Des..

[6] Agarwal A., Aponte-Mellado A., Premkumar B.J., Shaman A., Gupta S. (2012). The effects of oxidative stress on female reproduction: A review. Reprod. Biol. Endocrinol..

[7] Halliwell B., Gutteridge J.M.C. (2007). Reactive Species Can Be Poisonous, in Free Radicals in Biology and Medicine.

[8] Valentini J., Grotto D., Paniz C., Roehrs M., Burg G., Garcia S.C. (2008). The influence of the hemodialysis treatment time under oxidative stress biomarkers in chronic renal failure patients. Biomed. Pharmacother..

[9] Rocha J.B.T., Saraiva R.A., Garcia S.C., Gravina F.S., Nogueira C.W. (2012). Aminolevulinate dehydratase (d-ALA-D) as marker protein of intoxication with metals and other pro-oxidant situations. Toxicol. Res..

[10] Agarwal A., Gupta S., Sharma R.K. (2005). Role of oxidative stress in female reproduction. Reprod. Biol. Endocrinol..

[11] Lunardi-Maia T., Schuelter-Trevisol F., Galato D. (2014). Medication use during the first trimester of pregnancy: Drug safety and adoption of folic acid and ferrous sulphate. Rev. Bras. Ginecol. Obstet..

[12] Leal C.A., Schetinger M.R., Leal D.B., Morsch V.M., da Silva A.S., Rezer J.F., de Bairros A.V., Jaques J.A. (2011). Oxidative stress and antioxidant defences in pregnant women. Redox Rep..

[13] Silva L.S.V., Thiapó A.P., Souza G.G., Saunders C., Ramalho A. (2007). Micronutrients in pregnancy and lactation. Rev. Bras. Saude Matern. Infant..

[14] Devrim E., Tarhan I., Ergüder I.B., Durak I. (2006). Oxidant/antioxidant status of placenta, blood, and cord blood samples from pregnant women supplemented with iron. J. Soc. Gynecol. Investig..

[15] Lapenna D., Ciofani G., Pierdomenico S.D., Giamberardino M.A., Cuccurullo F. (2001). Reaction conditions affecting the relationship between thiobarbituric acid reactivity and lipid peroxides in human plasma. Free Radic. Biol. Med..

[16] Boyne A.F., Ellman G.L. (1972). A methodology for analysis of tissue sulfhydryl components. Anal. Biochem..

[17] Jacques–Silva M.C., Nogueira C.W., Broch L.C., Flores E.M., Rocha J.B. (2001). Dyphenyl disselenides and ascorbic acid changes deposition of selenium and brain of mice. Pharmacol. Toxicol..

[18] Galley H., Davies M.J., Webster N.R. (1996). Ascorbil radical formation in patients with sepsis: Effects of ascorbate loading. Free Radic. Biol. Med..

[19] Aebi H. (1984). Catalase *in vitro*. Meth. Enzymol..

[20] Berlin K., Schaller H. (1974). European standardized method for the determination of δ–aminolevulinic dehydratase activity in blood. Z. Klin. Chem. Klin. Biochem..

[21] Souza A.I., Filho M.B., Ferreira L.O.C. (2002). Hematological changes and pregnancy. Rev. Bras. Hematol. Hemoter..

[22] Hill C., Pickinpaugh J. (2008). Physiologic changes in pregnancy. Surg. Clin. N. Am..

[23] Cavalcante A.G.M., Bruin P.F.C. (2009). The role of oxidative stress in COPD: Current concepts and perspectives. J. Bras. Pneumol..

[24] Jacob R.F., Mason R.P. (2005). Lipid peroxidation induces cholesterol domain formation in model membranes. J. Biol. Chem..

[25] Lima E.S., Abdalla D.S.P. (2001). Lipid peroxidatiom: Mechanisms and evaluation in biological samples. Rev. Bras. Cienc. Farm..

[26] Chamy V.M., Lepe J., Catalán A., Retamal D., Escobar J.A., Madrid E.M. (2006). Oxidative stress is closely related to clinical severity of pre-eclampsia. Biol. Res..

[27] Hu M.L. (1994). Measurement of protein thiol groups and glutathione in plasma. Methods Enzymol..

[28] Yang Y., Guan X. (2015). Rapid and thiol–specific high–throughput assay for simultaneous relative quantification of total thiols, protein thiols, and non– protein thiols in cells. Anal. Chem..

[29] Suhail M., Patil S., Khan S., Siddiqui S. (2010). Antioxidant vitamins and lipoperoxidation in non–pregnant, pregnant, and gestational diabetic women: Erythrocytes osmotic fragility profiles. J. Clin. Med. Res..

[30] Hassan G.I., Onu A.B. (2006). Total serum vitamin C concentration in pregnant women: Implications for a healthy pregnancy. Rev. Bras. Saude Matern. Infant..

[31] Bloom J.C., Brandt J.T., Klaassen C.D. (2001). Toxic responses of the blood. Casarett and Doull’s Toxicology: The Basic Science of Poisons.

[32] Ahamed M., Siddiqui M.K.J. (2007). Environmental lead toxicity and nutritional factors. Clin. Nutr..

[33] Costa C.A., Trivelato G.C., Pinto A.M., Bechara E.J. (1997). Correlation between plasma 5–aminolevulinic acid concentrations and indicators of oxidative stress in lead–exposed workers. Clin. Chem..

[34] Gurer H., Ercal N. (2000). Can antioxidants be beneficial in the treatment of lead poisoning?. Free Radic. Biol. Med..

[35] Ademuyiwa O., Odusoga O.L., Adebawo O.O., Ugbaja R. (2007). Endogenous antioxidant defences in plasma and erythrocytes of pregnant women during different trimesters of pregnancy. Acta Obstet. Gynecol. Scand..

[36] Sauer E., Moro A.M., Brucker N., Nascimento S., Gauer B., Fracasso R., Gioda A., Beck R., Moreira J.C.F., Eifler–Lima V.L. (2014). Liver δ–aminolevulinate dehydratase activity is inhibited by neonicotinoids and restored by antioxidant agents. Int. J. Environ. Res. Public Health.

[37] Gabriel D., Pivetta L., Folmer V., Soares J.C., Augusti G.R., Nogueira C.W., Zeni G., Rocha J.B. (2005). Human erythrocyte δ–aminolevulinate dehydratase inhibition by monosaccharides is not mediated by oxidation of enzyme sulfhydryl groups. Cell Biol. Int..

[38] Ministry of Health Attention to Prenatal Low Risk. http://bvsms.saude.gov.br/bvs/publicacoes/cadernos_atencao_basica_32_prenatal.pdf.

[39] Abbaspour N., Hurrell R., Kelishadi R. (2014). Review on iron and its importance for human health. J. Res. Med. Sci..

[40] Grotto H.Z.W. (2010). Iron physiology and metabolism. Rev. Bras. Hematol. Hemoter..

[41] Casanueva E., Viteri F.E. (2003). Iron and oxidative stress in pregnancy. J. Nutr..

[42] Lachili B., Hininger I., Faure H., Arnaud J., Richard M.J., Favier A., Roussel A.M. (2001). Increased lipid peroxidation in pregnant women after iron and Vitamin C supplementation. Biol. Trace Elem. Res..

[43] Young B.C., Levine R.J., Karumanchi S.A. (2010). Pathogenesis of preeclampsia. Annu. Rev. Pathol..

[44] Garcia A.C., Roschel H., Ramos S., Benatti F.B. (2012). Iron supplementation and its association with the incidence of gestational diabetes mellitus. J. Braz. Soc. Food Nutr..

[45] Barreiros A.L.B.S., David J.M. (2006). Oxidative stress: Relations between the formation of reactive species and the organism’s defense. Quim. Nova.

[46] Han X.X., Sun Y.Y., Ma A.G., Yang F., Zhang F.Z., Jiang D.C., Li Y. (2011). NaFeEDTA, iron and oxidative stress in pregnancy. Asia Pac. J. Clin. Nutr..

[47] Kurtoglu E., Ugur A., Baltaci A.K., Undar L. (2003). Effect of iron supplementation on oxidative stress and antioxidant status in iron–deficiency anemia. Biol. Trace Elem. Res..

[48] Papas A.M. (1999). Diet and antioxidant status. Food Chem. Toxicol..

